# Why online science education falls short

**DOI:** 10.1016/j.isci.2025.113376

**Published:** 2025-08-16

**Authors:** Mohammed A. Mostajo-Radji

**Affiliations:** 1 Genomics Institute, University of California, Santa Cruz, Santa Cruz, CA 95064, USA; 2 Live Cell Biotechnology Discovery Lab, University of California, Santa Cruz, Santa Cruz, CA 95064, USA

**Keywords:** Computer science, Social sciences, Education

## Abstract

Remote science education has expanded rapidly, offering access to STEM learning at an unprecedented scale. Yet most online platforms prioritize content delivery through videos, simulations, and assessments, excluding the central experience of live experimentation. Without opportunities to generate data, test ideas, and engage in discovery, students miss chances to develop technical intuition, experimental reasoning, and ownership of the scientific process. This absence undermines inquiry-based learning, which builds understanding through doing rather than watching. Here, I examine how the current structure of online science education falls short, emphasizing the consequences of excluding real-time, hands-on investigation for reasoning, identity, and engagement. I highlight a growing class of cloud-connected platforms that allow students to remotely design, run, and analyze live experiments. By restoring access to real-time inquiry, these systems have been shown to improve learning outcomes and strengthen STEM identity, offering a promising direction for scalable, interactive science education that broadens participation globally.

## Graphical abstract

**Figure:**
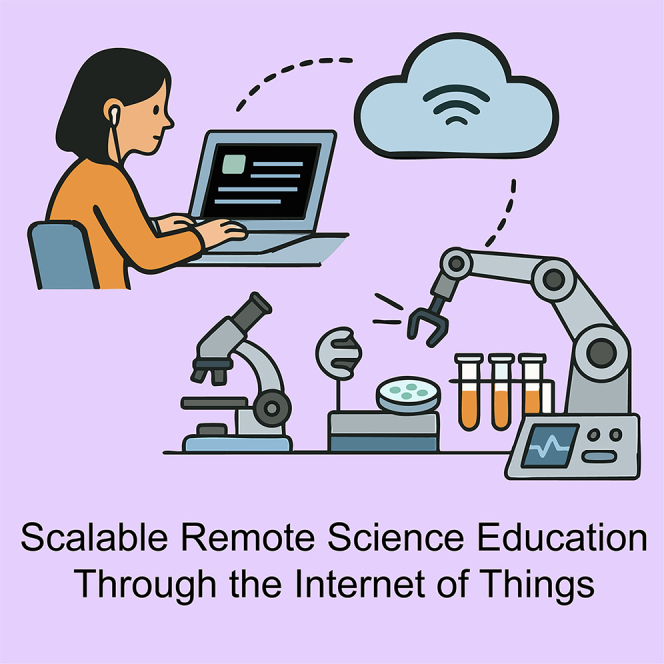

## Teaser

Computer science; Social sciences; Education; STEM education

## Introduction

Over the past two decades, online education has steadily expanded across disciplines.^1^ What began as a complement to in-person teaching has evolved into a core component of how science is taught and learned. This growth accelerated sharply during the COVID-19 pandemic, when institutions around the world were forced to adopt remote instruction at scale.^1^ In that moment, online platforms were not just a convenience; they became the default infrastructure for science education.

Since then, the digital classroom has become normalized. Educators have grown more comfortable designing remote materials, and students have grown more comfortable learning from them.^2^^,^^3^^,^^4^ Massive open online courses (MOOCs), learning management systems, and virtual lab modules are now embedded in STEM education at every level, from middle school to graduate programs.^5^^,^^6^^,^^7^ This shift has increased access to content and expanded the reach of science education across geographic and institutional boundaries.

Yet despite this progress, a critical element of science learning has been lost in the transition: the experiment.^8^^,^^9^^,^^10^ Most online science education models emphasize the transmission of information, not the process of discovery.^11^ They rely heavily on pre-recorded videos, simulations, and standardized assessments. These tools may be effective for teaching theory or reinforcing known outcomes, but they rarely offer students the chance to design, run, or interpret real experiments.^12^ As a result, students are often distanced from the core practices of scientific thinking.

Here, I examine why current models of online science education fall short, focusing on the absence of live experimentation. While the discussion centers on applications in wet-lab biology and engineering, the analysis highlights broader implications for science education wherever direct interaction with dynamic systems is essential. I also explore an emerging alternative: cloud-connected platforms that allow students to remotely engage with living systems in real time. These tools suggest a new path forward: one that restores the experimental core of science education while preserving the accessibility and scale of digital learning.

## The MOOC moment: A revolution that WASN'T

When MOOCs first emerged in the early 2000s, they were widely described as a revolution in education.^13^ These platforms promised to democratize access to university-level instruction by making high-quality courses available to anyone with an internet connection. In the sciences, institutions raced to upload lectures and design virtual labs that could reach thousands of learners at once. The vision was expansive. Education would be open, scalable, and free from the constraints of location or tuition.

While MOOCs successfully expanded access to content, they did not transform how science is taught or who benefits from it. Empirical studies soon revealed that the majority of MOOC participants already held college or graduate degrees and that completion rates were low among students without prior academic experience.^14^ Instead of closing educational gaps, MOOCs often reinforced them. Learners with strong preparation, reliable internet, and high digital literacy were most likely to succeed. Others struggled to engage meaningfully or persist through the course.^15^

These limitations are part of a broader pattern in online education. Digital learning environments can reinforce three types of inequality: vital inequality in the quality of educational experiences, resource inequality in access to tools and infrastructure, and existential inequality in the visibility and agency afforded to learners.^16^ In most MOOCs, students consume polished content but have little opportunity to shape the learning process or contribute to the creation of knowledge. They are often treated as recipients rather than participants.

This disconnect is particularly damaging in science education. Scientific understanding is not only conveyed through concepts but developed through experimentation and iteration.^17^^,^^18^ In most MOOCs, students do not generate data, manipulate living systems, or engage with real uncertainty. As a result, they miss the chance to develop technical intuition and experimental reasoning. The version of science they experience is static, observational, and detached from practice.

The shortcomings of MOOCs do not invalidate the potential of online science education.^15^ Rather, they point to the need for new models that retain the scale and flexibility of digital platforms while restoring the hands-on, inquiry-driven character of scientific learning.

## The educational cost of missing experimentation

The absence of live experimentation in online science education has measurable consequences for learning outcomes, motivation, and identity formation. While virtual tools can help convey concepts, they often fail to support the full development of scientific reasoning and hands-on intuition.

A study of undergraduate chemistry students found that those participating in physical laboratory work significantly outperformed students assigned to virtual-only conditions. Post-test achievement and laboratory report quality were both higher in the hands-on group, demonstrating the limits of simulation-based learning.^19^ Similarly, in a controlled study in molecular biology, students using scenario-based simulations showed improvements in knowledge, motivation, and self-efficacy, but these gains still lacked the depth associated with working directly with biological materials.^20^

In physics education, students who engaged in real-world beam-bending experiments performed similarly to those using remote labs when solving basic problems. However, the hands-on group outperformed peers when faced with more complex tasks, and they rated their experience as significantly more immersive and intellectually engaging.^21^ This suggests that physical experimentation provides not only cognitive benefits but also a stronger emotional and attentional connection to the material.

Large-scale reviews have further demonstrated the value of active engagement in science learning. A meta-analysis of 225 STEM studies showed that active learning approaches, which include inquiry and experimentation, reduced failure rates from 33.8% to 21.8% and improved conceptual understanding by nearly half a standard deviation.^22^ In another study, students in redesigned physics labs emphasized data-driven thinking were twelve times more likely to improve their methods and four times more likely to question flawed models, compared to those in traditional settings.^23^

These findings point to a consistent pattern. Without direct experimentation, students may learn scientific content, but they do not learn to think, act, or problem-solve like scientists. They are not given opportunities to make mistakes, test hypotheses, or adapt their strategies based on outcomes. The scientific process becomes something they observe, rather than something they perform. This undermines the formation of core skills and deprives students of the confidence that emerges through doing.

## Technology access is not enough

Like MOOCs, the One Laptop per Child (OLPC) initiative began with widespread optimism. The project aimed to transform education by giving low-cost laptops to children around the world.^24^ Its core assumption was that access to digital devices would unlock creativity, increase motivation, and improve learning outcomes.^24^

Studies from Latin America and beyond challenge that assumption. In Peru, a randomized trial in low-income schools found that while students improved their computer proficiency, there were no gains in academic achievement or cognitive skills. Teachers also reported a decline in student effort.^25^ A 10-year follow-up across 500 rural schools confirmed that the program had no long-term effect on academic progress despite increased familiarity with technology.^26^ A related national deployment showed no improvement in language or mathematics test scores.^27^

In Uruguay, the Ceibal program successfully distributed laptops to all public school students and received strong public support. However, evaluations reported little to no impact on literacy, numeracy, attendance, or classroom engagement.^28^ In Brazil, a national review of the OLPC adaptation found that without teacher training and pedagogical planning, the program failed to improve learning outcomes despite heavy investment in infrastructure.^29^ Technical challenges and underutilization further reduced the educational value of the devices.^30^

In Southeast Asia, similar challenges emerged. Thailand’s One Tablet Per Child (OTPC) policy faced implementation problems, particularly in rural areas, where lack of localized content and insufficient teacher support limited its effectiveness.^31^ A related study found that tablets sometimes increased not only student interest but also teacher workload without improving academic outcomes.^32^ In Cambodia, digital device exposure among young children showed no meaningful cognitive benefits once household and background factors were taken into account.^33^

These patterns also appear in sub-Saharan Africa. In Kenya and Rwanda, laptop initiatives produced learning gains only when combined with curriculum-aligned instruction and active teacher involvement.^34^^,^^35^^,^^36^^,^^37^ In South Africa, rural technology programs showed similarly limited academic impact, as tablets were often poorly integrated into lessons and undercut by connectivity issues and lack of pedagogical support.^38^

These examples reveal a consistent finding. Technology alone does not change how students learn. Without inquiry-based instruction, teacher engagement, and clear learning goals, devices become passive tools.^39^ This is especially true in science education, where learning depends on experimentation, problem solving, and interpreting results.^17^ Infrastructure can increase access but cannot replace the active process of learning through doing.

## Simulations are not scientific inquiry

Online science education has increasingly relied on lab simulations to approximate experimental work.^40^ These digital platforms allow students to mix reagents, collect measurements, and observe results in structured environments. Simulations are scalable, cost-effective, and often improve conceptual understanding when used alongside guided instruction.^41^^,^^42^

Despite these strengths, simulations fall short of enabling true scientific inquiry.^43^ Real experimentation is characterized by unpredictability, noisy measurements, failed setups, and the need for iterative troubleshooting. These elements cultivate reasoning, adaptability, and hypothesis-driven thinking. Simulations, in contrast, typically follow predetermined workflows where variables behave as expected and results are preprogrammed.^44^^,^^45^

Empirical comparisons underscore this point. A study of Indonesian high school students found that while both physical and virtual pendulum labs improved physics knowledge, only the students in real labs demonstrated gains in designing follow-up experiments and evaluating error sources.^46^ A broader review confirmed that while simulations support theory acquisition, they do not foster procedural decision-making or investigative autonomy.^45^ Even in technical fields like cybersecurity, where virtual labs are widely adopted, the outcomes remain mixed. In a study evaluating simulation-based cybersecurity training, only a minority of students reported that virtual labs helped improve their active engagement or problem-solving abilities. While the structured exercises supported familiarity with concepts, most learners struggled to transfer these skills to open-ended or real-world scenarios.^47^

Feedback from students also points to these limitations. A 2023 survey of over 1,600 learners and instructors found that while most valued the accessibility of virtual labs, the majority reported feeling underprepared to conduct real investigations and expressed a desire for more interactive components.^48^

One example of a more flexible virtual platform is LabXchange. Unlike conventional simulations, LabXchange allows students and instructors to assemble modular learning pathways, combining videos, protocols, assessments, and interactive tools. Its flexibility supports inquiry-based learning and has been linked to higher student motivation and conceptual gains in both Latin American and Southeast Asian classrooms.^49^^,^^50^^,^^51^^,^^52^ However, LabXchange still relies on scripted simulations and fixed outputs. It does not allow students to operate instruments, collect live data, or encounter unexpected behaviors. It serves as a pedagogical scaffold rather than a platform for experimentation.

Simulations also come with technical requirements that can limit access. Many demand stable internet, high browser performance, and recent operating systems to support three-dimensional rendering or real-time interactions. These demands pose challenges for students in low-resource environments, where access to high-performance devices and broadband connectivity remains uneven.^53^^,^^54^ Institutions also face implementation barriers such as licensing fees and platform integration needs, especially when support staff are limited.

In summary, simulations can effectively convey theory and scaffold learning,^55^ but they do not foster the skills, reasoning, or open-ended mindset associated with scientific inquiry. Their limitations are not merely pedagogical but structural. To bring inquiry back into remote science education, new approaches are needed that allow students to design, execute, and interpret experiments in real time, even when not physically present.

## Remote instruction allows hands-on science at home but not without barriers

Hands-on laboratory courses, including those delivered remotely, have played a central role in inquiry-based science education, fostering experimental design, autonomy, and critical thinking.^56^^,^^57^ The COVID-19 pandemic accelerated efforts to reimagine remote lab instruction, prompting the deployment of distributed, at-home experiments across disciplines. These efforts also exposed persistent logistical and pedagogical challenges that remain relevant beyond emergency instruction.^58^

Several programs developed experimental kits that could be mailed to students for synchronous and asynchronous instruction. A remote general chemistry course for 800 students used pre-assembled kits distributed through university bookstores. The kits replaced precision glassware with accessible alternatives and included Arduino-based spectroscopy. Students reported a strong sense of connection to the experimental process, and the centralized distribution ensured equity in access.^59^ Another program adapted chemistry experiments for the kitchen, reducing chemical hazards while preserving core learning goals. In that study, 88% of students agreed that the activities helped them understand the material, and many reported that the accessibility of materials increased their confidence in conducting experiments independently.^60^

Some programs asked students to source their own materials. A high school biotechnology course implemented open-ended antibacterial testing using household items. While students appreciated the freedom to explore personal hypotheses, instructors noted large variation in project complexity, raising concerns about comparability and equitable outcomes.^61^ In a comparative study of three remote chemistry formats, students who participated in hands-on formats, whether through mailed kits or hybrid demonstrations, showed higher engagement and academic performance than those in fully virtual formats.^62^

Remote laboratory approaches varied in cost and complexity. A neuroscience course for undergraduates shipped open-source SpikerBox kits that enabled students to collect EEG, ECG, and EMG data from their own bodies. Equipment costs ranged from $150 to $400 per student. Students who performed experiments at home showed significantly larger improvements in self-efficacy and confidence in their scientific skills compared to peers who only watched videos.^63^ Similarly, a remote genetics course used a CRISPR kit to allow students to perform yeast-based gene editing. Despite its higher cost (over $200 per kit), the activity was widely praised by students for offering authentic molecular biology training.^64^ These results mirror findings from a Latin American program that implemented classroom-based CRISPR training with secondary students. The study found significant gains in motivation and conceptual understanding, even in schools with limited lab resources.^57^

Not all implementations required mailing kits. A chemical kinetics experiment based on the Fenton reaction allowed students to conduct experiments at home using hydrogen peroxide and iron salts. Students who had access to materials achieved learning outcomes comparable to those in traditional settings. However, motivation declined after the activity, particularly among those who struggled to obtain materials or follow procedures independently.^65^

These studies demonstrate the potential of remote laboratory education to provide rigorous, inquiry-based experiences. However, they also expose logistical and equity-related barriers. Kit-based instruction involves cost and coordination challenges, particularly when equipment exceeds $200 per student. While some courses achieved scale through partnerships with bookstores or shared distribution centers, others faced difficulties reaching students in time. Additional barriers include assumptions about home access to everyday tools like thermometers, power outlets, or heating elements. The most successful implementations addressed these challenges by providing optional datasets for students unable to collect their own, offering guidance through asynchronous and synchronous formats, and minimizing hardware and internet demands.^66^

Remote laboratories are not inherently inferior to in-person labs, but their success depends on thoughtful design, accessible infrastructure, and logistical coordination. Without these, efforts to scale hands-on science education risk reinforcing the very inequalities they aim to overcome.

## Live biology at a distance: Cloud labs for scalable, hands-on learning

**Figure 1:**
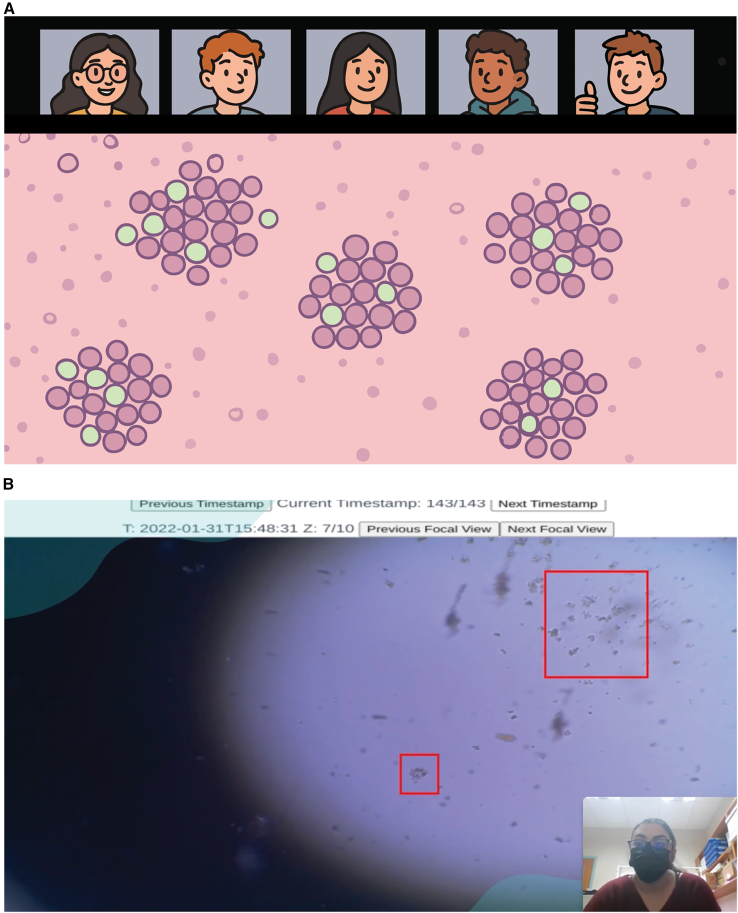
Example of a course implementing cloud labs (A) Caricature of remote learning interactions during cloud-based experiments. Students meet on a videoconferencing platform such as Zoom or Microsoft Teams, where they collaboratively design experiments and discuss real-time manipulations. The live experiments are streamed to students using platforms like YouTube, Twitch, or custom-built interfaces, with YouTube often preferred in low-bandwidth regions due to its adaptive streaming capabilities. Students can program experimental protocols for execution and, after class, continue to monitor and manipulate experiments independently using personal devices such as phones, tablets, or computers. After the project is completed, students reconvene to analyze results and discuss their findings and may share outcomes through reports, videos, or posters. (B) Student interacting with live cell cultures via cloud microscopy using the Picroscope platform.

Cloud laboratories are reshaping how biology is taught in remote and distributed settings. These platforms allow students to conduct real-time, live-cell experiments using internet-connected devices to control microscopes, robotic systems, and biosensors from anywhere in the world^67^ (Figure 1). Sitting between home-based kits and virtual simulations, cloud labs provide the authenticity of live experiments and the scalability of digital education (Table 1). Unlike kits, they do not require shipment of physical materials or direct handling of reagents. Unlike virtual labs, they allow students to engage with real biological variability, collect live data, and develop scientific reasoning through open-ended inquiry.

**Table 1:** Comparison of remote science education modalities

Modality	Example platforms or models	Real-time data generation	Requires physical materials	Enables discovery-based learning	Scalability	Technical requirements
MOOCs	Coursera, EdX	no	no	no	very high	low (web browser)
Virtual Labs	LabXchange, Labster	no	no	no	high	moderate (browser, GPU optional)
At-Home Kits	CRISPR kits,^64^ Backyard Brains kits^63^	yes	yes	yes	limited by logistics	moderate to high (shipping, setup)
Cloud Labs	Picroscope,^56^ Streamscope^68^	yes	no	yes	very high	low to moderate (internet, device)

The earliest cloud lab efforts to reach scale came from the work of Hossain and colleagues, who demonstrated that students could perform interactive biological experiments remotely with real-time control and observation. In one foundational study, students manipulated the phototactic behavior of *Euglena gracilis* by adjusting light direction through cloud-connected microfluidic chips.^69^ This platform was later scaled to support over 2,300 experiments conducted by students in more than 40 countries, providing some of the first empirical evidence that cloud experimentation could be reliable, reproducible, and pedagogically impactful across continents.^70^

Since then, cloud labs have diversified in both complexity and scope. Systems like the Picroscope allow for continuous imaging of multicellular cultures, including neuroblastoma cells and zebrafish embryos.^71^ The Picroscope features 24 Raspberry Pi cameras positioned over multiwell plates, enabling students to collect longitudinal data from multiple samples at once. In the Salinas Valley of California, students used the system to explore the toxic effects of agricultural fertilizers on development. In Latin America, cloud labs have been used to investigate public health questions, such as the biological safety of chlorine dioxide, a chemical misrepresented in public discourse.^72^ These modules enabled students to carry out original investigations using real-time data streams and discuss findings with peers and instructors.^56^

The Streamscope, a complementary platform, uses adaptive streaming algorithms and public video platforms like YouTube to deliver live microscopy feeds directly to students.^68^ Because it requires no special software installation and performs well even under limited bandwidth conditions, the Streamscope has made it possible to deploy live-cell education modules in regions with unreliable internet infrastructure.^67^

Beyond microscopy, cloud labs support experimental control through robotic systems such as EvoBot and OpenLH. These platforms automate liquid-handling tasks such as pipetting and reagent mixing.^73^^,^^74^ Their lightweight software interfaces allow students to queue actions and monitor workflows, enabling full experimental cycles including sample preparation, treatment, and observation. These tools have been used in courses focusing on synthetic biology, microbial growth dynamics, and even biological art.

Electrophysiological cloud platforms expand access to neural experimentation. PiPhys allows students to record extracellular signals from cultured neurons using a low-cost amplifier system.^75^ The MaxOne, a high-density multielectrode array with over 26,000 electrodes per well, has been adapted for cloud-based educational use with brain organoids.^68^ These systems offer rich datasets on spiking activity and network dynamics, connecting students to advanced concepts in neurobiology and data science.

Lab-on-a-chip platforms further extend the range of biology that can be taught through cloud labs. In one course, students used an internet-controlled microfluidic device to detect bacterial contamination in water samples using fluorescent DNA dyes.^76^ Without any prior programming experience, students were able to modify code, control valves remotely, and analyze real-time optical output. This integration of biology and computer science shows how cloud labs can promote interdisciplinary education and coding literacy.

One of the most significant pedagogical advantages of cloud labs is their ability to support authentic scientific inquiry. Students must generate hypotheses, interpret live data, and respond to unexpected results, mirroring the conditions of real research. This is in contrast to virtual labs, where learning outcomes are scripted and uncertainty is removed. Instructors have also reported that when students work on projects tied to local environmental or social issues, engagement improves and STEM identity is strengthened.^56^^,^^57^

Importantly, cloud labs do not require detailed engineering knowledge on the part of the user. All the complexity of instrument control and data collection is handled by lightweight, modular software designed for educational use. Students can perform experiments, visualize results in real time, and export data for quantitative analysis using open tools like ImageJ or Python.^77^ These platforms have also been architected for central operation, meaning schools do not need to maintain equipment locally.^77^ This allows for efficient scaling across institutions with varying levels of infrastructure.

Adaptive streaming algorithms, such as those used in Streamscope, ensure that access is preserved even in bandwidth-limited environments. Because data rates adjust automatically, cloud labs can operate over mobile networks or in regions with unstable connections. When combined with intuitive interfaces,^78^ this capability helps make experimental biology accessible to students in rural or under-resourced communities.

Cloud labs are not only technical innovations but also pedagogical ones. They reintroduce scientific exploration into online education, enabling students to ask questions, manipulate systems, and interpret live outcomes. In doing so, they restore an essential element of science education that is often missing from remote instruction: the opportunity to engage with the unexpected. As adoption grows, cloud labs may become the backbone of a new kind of biological education: scalable, inquiry-driven, and inclusive.

## Cognitive and contextual dimensions of cloud lab learning

Cloud-connected biology labs represent a distinct mode of remote experimentation that enables live, student-driven interaction with biological systems. Unlike preprogrammed virtual labs or passive video instruction, cloud labs support a range of engagement types depending on how students interact with the platform and with each other. To classify these differences, I apply the ICAP framework, which defines four modes of cognitive engagement: Interactive, Constructive, Active, and Passive.^79^^,^^80^ These modes are ordered from highest to lowest cognitive demand and learning outcome:

- Interactive engagement occurs when learners work collaboratively and build on one another’s ideas in a co-generative way.
- Constructive engagement involves learners generating new knowledge beyond what was provided, such as formulating hypotheses or drawing inferences.
- Active engagement requires selecting or manipulating information without generating new content, such as clicking through a simulation or executing predefined protocols.
- Passive engagement is characterized by the reception of information without any observable response, such as watching a video.

Table 2 applies this framework to common modalities in remote science education. Standard online courses and simulations are typically Passive. Remote lab kits often fall into the Active category, providing hands-on access but limited opportunities for students to create or revise experimental designs. Cloud labs can support any ICAP level depending on their instructional design. When students remotely control experiments and receive dynamic feedback, they engage at the Constructive level; embedded in collaborative workflows, these activities reach fully Interactive engagement.

**Table 2:** Cognitive engagement levels across remote science education modalities (ICAP framework)

Modality	Passive	Active	Constructive	Interactive	Typical learner action
MOOCs	✓	–	–	–	watching lecture videos
Virtual labs	–	✓	✓	–	manipulating variables to observe outcomes
Remote kits	–	✓	–	–	following pre-designed hands-on protocols
Simulations	–	✓	✓	–	testing scenarios and interpreting results
Cloud labs	–	✓	✓	✓	designing and modifying live experiments with peer feedback

A check mark indicates that the modality is capable of attaining this level of engagement when the appropriate instructional design and support are provided.

One study showed that students from different countries co-designed and executed live experiments through a shared cloud lab platform, making predictions, modifying protocols, discussing outcomes, and revising interpretations together.^56^ The activity supported generative, co-constructed engagement consistent with the highest ICAP level.

Feedback mechanisms and interface design play a critical role in supporting deeper forms of engagement. Platforms that enable students to vary inputs, visualize real-time results, analyze outcomes, and adjust parameters in response promote Constructive engagement. When these systems also support asynchronous coordination, shared access to results, or collaborative annotation tools, they provide a foundation for Interactive learning.^77^^,^^81^ In contrast, cloud platforms that require students to follow a rigid sequence without the opportunity for decision-making or reflection are more likely to result in Active or Passive learning, even if they involve live biological samples.

What sets cloud labs apart from other remote science approaches is their ability to restore authentic experimentation and critical thinking to online education.^67^ The ICAP framework makes it possible to evaluate how students engage cognitively, based on what they do rather than what content is delivered. Designing cloud lab activities that support student-led exploration, collaborative reasoning, and iteration can help move beyond traditional remote instruction toward deeper, more authentic learning experiences. Realizing the full potential of Interactive engagement, however, depends not only on the technical capabilities of the platform but also on how cloud labs are embedded within structured, collaborative learning environments.

In addition to platform features and collaboration structures, cultural and contextual relevance also shape how students engage with cloud labs. Instructional design that connects science learning to students’ cultural context and lived experience has been shown to improve engagement, interpretation, and analytical reasoning. Integrating regional biodiversity and traditional knowledge into biology modules can enhance higher-order thinking, even without advanced infrastructure.^82^ Similarly, inquiry-based group activities that emphasize iteration and peer interaction can support gains in problem-solving and scientific reasoning, even in resource-limited settings.^8^ These principles have been applied in cloud-based classrooms where students explored bacterial contamination in local water sources using real-time experimental platforms. The activity linked scientific inquiry with community concerns and reinforced the relevance of experimental biology and computational tools.^76^ Together, these cases highlight how cloud labs can serve as context-aware platforms that support culturally grounded, inquiry-driven learning at scale.

## Educational outcomes of cloud labs

Cloud labs have been implemented in high schools, colleges, and advanced educational programs such as the National Youth Science Camp and Science Clubs International.^56^ Their educational impact has been assessed along two key dimensions: knowledge gained and changes in STEM identity.

In terms of knowledge acquisition, a study compared college students in Bolivia who completed either an in-person or a cloud-lab-based inquiry course using matched curricula. The cloud lab group (*n* = 17) and in-person group (*n* = 24) were tested on the scientific method, experimental design, data analysis, and outlier detection. Both groups performed similarly across these assessments. These results demonstrate that remote, cloud-lab-enabled inquiry can achieve learning outcomes that are comparable to those of traditional in-person laboratory education.^56^

The same study evaluated changes in STEM identity using two validated instruments: the single-item STEM Professional Identity Overlap^83^ (STEM-PIO-1) and the more comprehensive Role Identity Survey in STEM^84^ (RIS-STEM), which measures competence, interest, self-recognition, and recognition by others. High school students in the United States who used cloud labs in biology classrooms showed significant gains in STEM identity. When the RIS-STEM was applied to students in both the United States and Bolivia who had undergone the same intervention, the study revealed a larger increase in STEM interest among Bolivian students.^56^ This direct cross-national comparison, made possible by cloud labs, is rarely feasible in educational research and highlights the potential of this approach for scalable and globally relevant STEM education.

Cloud labs have also been used to bridge disciplines. In a college mathematics course, students interacted with live brain organoid experiments via a cloud-connected brain-machine interface. Among the 24 students, 83.3% reported increased interest in stem cells, 87.5% in organoids, and 87.5% indicated they would consider a career in neuroscience or stem cell research.^68^ These results closely matched outcomes from interventions in biology courses.^68^ This shows the ability of cloud labs to extend authentic scientific engagement beyond traditional life science classrooms and into new academic domains.

## Discussion

The rapid expansion of remote science education has increased global access to instructional content but has also exposed a persistent gap between learning about science and doing science. Most platforms have focused on the delivery of knowledge through videos, simulations, and assessments while leaving behind the essential element of experimentation. As a result, students often gain conceptual understanding without developing procedural fluency, critical reasoning, or a sense of scientific agency.

Some remote learning models have demonstrated that deep cognitive engagement is possible without physical experimentation. One example is a biochemistry course where students authored and refined multiple-choice questions as part of a structured peer-learning system that encouraged generative and collaborative thinking at scale.^85^ While this approach highlights the potential of remote instruction to support higher-order learning, it also illustrates that such engagement depends on intentional design. The same is true for cloud labs. Their ability to foster inquiry, reasoning, and scientific agency depends not just on providing access to live systems but on how these experiences are embedded within structured learning communities. Collaborative frameworks can be effectively paired with cloud-connected experimentation to help students bridge practical experiences with conceptual understanding.^56^^,^^67^^,^^85^ When implemented together, these approaches could support both hands-on and minds-on learning in scalable, inclusive formats.

This article highlights a consistent finding across studies and disciplines: engagement with live, hands-on experimentation is a key driver of motivation, comprehension, and skill development in science education.^86^ While the focus here is on applications in wet-lab biology and engineering, similar strategies are emerging across other fields. For example, cloud-based soil monitoring systems have been used in hybrid forestry and plant science courses in Israel and Spain, allowing students to remotely track plant growth and environmental conditions.^87^^,^^88^ These courses have achieved learning outcomes similar to those seen with cloud-enabled wet-lab experiments. In ecology, GPS-enabled tracking chips implanted in migratory birds and marine mammals provide real-time data on animal behavior and movement.^89^ When integrated into coursework, these tools could allow students to develop ecological hypotheses, analyze live telemetry data, and investigate environmental patterns, extending the reach of cloud-connected experimentation beyond the laboratory^90^^,^^91^^,^^92^.

Whether comparing kitchen-based chemistry experiments,^60^ CRISPR training modules,^64^ or cloud-enabled neuroscience labs,^68^ the pattern remains clear. When students are invited to ask questions, make decisions, and interpret messy data, they begin to think and act like scientists.

Simulations and digital modules, while valuable for reinforcing theory and scaling content,^40^ cannot fully replace the uncertainty and adaptation involved in real experimentation. Even the most interactive platforms tend to follow scripted paths that reduce the opportunity for discovery. Without challenges like failure, ambiguity, or surprise, students miss opportunities to build resilience, creativity, and scientific intuition.

Attempts to address these limitations through at-home kits have shown promise but face structural constraints related to cost, coordination, and accessibility.^57^ Many students remain excluded from these experiences due to shipping logistics, financial barriers, or lack of household resources. The result is a fragmented and uneven landscape where only some students gain meaningful exposure to the practices of science.

Cloud labs offer a compelling alternative.^67^ By providing remote access to real instruments, live cells, and data-rich experiments, they bring scientific inquiry within reach of geographically distributed and resource-constrained classrooms. They remove the logistical barriers of physical kits and the epistemic constraints of simulations while supporting authentic engagement with biological complexity.

Importantly, cloud labs are not just technical solutions. They represent a pedagogical shift toward restoring experimentation as the foundation of science education. By allowing students to engage with live systems, modify parameters, and explore unexpected outcomes, they reintroduce the core principles of inquiry.^67^ These tools also align with broader efforts to make science more inclusive.^56^^,^^67^

Online science education is not inherently flawed, but its current emphasis on passive learning constrains its transformative potential. Cloud-connected experimental platforms show that it is possible to scale access while preserving the hands-on, minds-on spirit of science. The next generation of learners should not only watch science happen but do it themselves, in real time, from wherever they are.

## Acknowledgments

This work was supported by the following grants: Schmidt Futures (SF857), the National Human Genome Research Institute (RM1HG011543), the National Institute of Mental Health (U24MH132628), the California Institute for Regenerative Medicine (DISC4-16285 and DISC4-16337), the University of California Office of the President (M25PR9045), and the University of California Santa Cruz Center for Information Technology Research in the Interest of Society and the Banatao Institute Interdisciplinary and Innovation Program (I2P).

## Declaration of interests

The author declares no competing interests.

## Declaration of generative AI and AI-assisted technologies in the writing process

During the preparation of this work, the author utilized ChatGPT to enhance language clarity and readability. All content was subsequently reviewed and edited as needed, and the author takes full responsibility for the final publication.
